# Hepatitis C Virus NS3 Mediated Microglial Inflammation via TLR2/TLR6 MyD88/NF-κB Pathway and Toll Like Receptor Ligand Treatment Furnished Immune Tolerance

**DOI:** 10.1371/journal.pone.0125419

**Published:** 2015-05-12

**Authors:** Ayilam Ramachandran Rajalakshmy, Jambulingam Malathi, Hajib Naraharirao Madhavan

**Affiliations:** 1 L & T Microbiology Research Centre, Vision Research Foundation, Chennai, India; 2 Centre for Nanotechnology and Advanced Biomaterials, SASTRA University, Thanjavur, India; Indiana School of Medicine, UNITED STATES

## Abstract

**Background:**

Recent evidence suggests the neurotrophic potential of hepatitis C virus (HCV). HCV NS3 protein is one of the potent antigens of this virus mediating inflammatory response in different cell types. Microglia being the immune surveillance cells in the central nervous system (CNS), the inflammatory potential of NS3 on microglia was studied. Role of toll like receptor (TLR) ligands Pam2CSK3 and Pam3CSK4 in controlling the NS3 mediated microglial inflammation was studied using microglial cell line CHME3.

**Methods:**

IL (Interleukin)-8, IL-6, TNF-α (Tumor nicrosis factor alpha) and IL-1β gene expressions were measured by semi quantitative RT-PCR (reverse transcription-PCR). ELISA was performed to detect IL-8, IL-6, TNF-α, IL-1β and IL-10 secretion. FACS (Flourescent activated cell sorting) was performed to quantify TLR1, TLR2, TLR6, MyD88 (Myeloid differntiation factor 88), IkB-α (I kappaB alpha) and pNF-κB (phosphorylated nuclear factor kappaB) expression. Immunofluorescence staining was performed for MyD88, TLR6 and NF-κB (Nuclear factor kappaB). Student's t-test or One way analysis of variance with Bonferoni *post hoc* test was performed and p < 0.05 was considered significant.

**Results:**

Microglia responded to NS3 by secreting IL-8, IL-6, TNF-α and IL-1β via TLR2 or TLR6 mediated MyD88/NF-κB pathway. Transcription factor NF-κB was involved in activating the cytokine gene expression and the resultant inflammatory response was controlled by NF-κB inhibitor, Ro106-9920, which is known to down regulate pro-inflammatory cytokine secretion. Activation of the microglia by TLR agonists Pam3CSK4 and Pam2CSK4 induced immune tolerance against NS3. TLR ligand treatment significantly down regulated pro-inflammatory cytokine secretion in the microglia. IL-10 secretion was suggested as the possible mechanism by which TLR agonists induced immune tolerance. NS3 as such was not capable of self-inducing immune tolerance in microglia.

**Conclusion:**

In conclusion, NS3 protein was capable of activating microglia and the inflammatory response could be controlled via blocking the transcription factor NF-κB, or by treating the microglia with TLR ligands which likely function via secreting anti-inflammatory cytokines such as IL-10. This can have therapeutic potential in controlling HCV mediated neuroinflammation.

## Background

Hepatitis C virus (HCV) is a RNA virus from *Flaviviridae* family [1]. HCV primarily infects liver which can induce hepatocellular carcinoma and liver cirrhosis [2]. Recent evidence points the neuroinvasion of HCV most probably via "Trojan Horse" mechanism [3]. This postulate is evident by PCR as well as immunohistochemical studies on clinical specimens. The viral RNA has been detected from the post mortem brain tissue [4]. Viral replication intermediates have been detected from the cerebrospinal fluid [5]. Several studies shows that HCV RNA sequences derived from liver and brain differ phylogenitically indicating the emergence of mutant forms of the virus which could replicate in the brain [6, 7]. The expression of HCV receptors such as scavenger receptor class B type I (SR-B1), tetraspanin CD81 and tight junction proteins, claudin-1 and occludin in the microglia and astrocytes indicates the possible viral entry into these cells [8–10]. HCV RNA encodes for a single poly protein which is cleaved by viral and host proteases to form structural and non-structural proteins [11]. HCV NS3 is a non-structural protein which has protease as well as helicase activities [12]. Current studies have revealed the antigenic potential of this protein, as NS3 is known to activate inflammatory pathways in monocytes [13] and anti-NS3 antibodies has been detected in the HCV positive patient sera [14]. Also astrocytes and perivascular macrophages in the brain sections from HIV/HCV coinfected patients were positive for HCV NS3 [15].

Toll like receptors (TLR) are pattern recognition receptors present on many cell types which participate in innate immune response associated with viral infections and viral antigens [16]. MyD88/NF-kB mediated cell signalling is one of the common pathways engaged in the inflammatory response with respect to TLR activation [17]. HCV NS3 is known to mediate inflammation via TLR2 in monocytes [13]. Microglia express functionally active TLR receptors, TLR1 to TLR9 [18]. The role of TLR participation in mediating the immune response could be bidirectional, regulated activation of TLRs is found to be protective in neuroinflammation at the same time this mechanism could be cytotoxic [19]. Immune activation by toll like receptor agonists has been implemented in therapeutic regimes, pulmonary administration of phospholipid conjugated TLR7 was found to be protective against different infectious agents by stimulating local immune response in mice models [20], various TLR agonists were used as vaccine adjuvants for immunotherapies [21] and for experimental cancer therapies [22].

The current study demonstrates the immune response of microglia against HCV NS3 antigen. Recombinant HCV NS3 protein induced inflammatory cytokine secretion in microglia by TLR2 or TLR6 mediated NF-kB signalling pathway. Inhibition of the NF-kB activation down regulated the inflammatory response. Pre treatment of the microglia with two synthetic TLR agonists Pam2CSK4 and Pam3CSK4 induced immune tolerance against NS3 mediated inflammation via IL-10 secretion. This is the first study demonstrating the microglial immune response against HCV NS3. Our results revealed the specific TLR participation in mediating inflammation which could be detrimental and the same response could be utilized for the immune protection against NS3 mediated microglial inflammation. Thus TLR response can be bifunctional and the TLR agonist mediated immune protection has therapeutic role in controlling HCV associated neuroinflammation.

## Materials and Methods

### Ethics statement

The study was approved by the Ethics Committee of the institute (Vision Research Foundation, Sankara Nethrlaya).

### Cell culture

SV40 immortalized human microglial cell line CHME3 established from human embryonic macrophages [23] was used in the present study (a kind gift from Dr. Anirban Basu, National Brain Research Centre, Haryana, India). The cell line was positive for CD11b and negative for GFAP at the transcript level (S1 Fig). CHME3 cells were cultured and maintained in Dulbecco's modified Eagle's medium (Gibco, Carlsbad, CA) and antibiotics (100 units/ml penicillin G and 100 units/ml streptomycin (Invitrogen, Carlsbad, CA)) with 10% fetal bovine serum (Gibco, Carlsbad, CA). Incubated at 37°C in a humidified incubator with 5% CO_2_.

### Chemicals and reagents

Recombinant HCV NS3 was purchased from Sigma (St Louis, MO). NF-kB inhibitor Ro 106–9920 was purchased from Santacruz biotechnology (Santacruz, CA). Pam2CSK4 and Pam3CSK4 were purchased from Invivogen (Santiago, CA). All the primary antibodies used in the study were from Santacruz Biotechnology (Santacruz, CA) and FITC conjugated secondary antibodies were from Dako (Denmark). Predesigned siRNAs for TLR6 (Catalog number: SI03112011) and negative control SiRNA (Catalog number: 1027280) were purchased from Qiagen, (Germany).

### RNA extraction and RT PCR

After different treatment conditions, total cellular RNA was extracted using Qiagen RNAse mini kit, 5μg of total RNA was used for cDNA conversion using sensiscript-RT kit (Qiagen, Hilden, Germany) and oligo-dT primers (Fermentas, USA). RT-PCR was performed for TLR1, TLR2, TLR6, IL-8, IL-6, TNF-α, IL-1β and GAPDH using equal amount of cDNA. All the PCR reagents were purchased from Fermentas, USA unless other than stated, all the primers except GAPDH were from Hysel biotech, India and GAPDH primers were from Bangalore Genei, India, all the primers and the product sizes are listed in table 1. PCR cycling conditions were as follows, 95°C for 10 minutes, followed by 35 cycles of 94°C for 1 minute, 60°C for 1 minutes for all the cytokine genes and 63°C for GAPDH and 72°C for 1 minutes, with a final extension of 72°C for 10 minutes. Equal volume of the PCR products were loaded on a 2% agarose gel with 0.5 μg/ml ethidium bromide and images were captured.

**Table 1:** RT-PCR primer list.

Gene	Primer	Size (bp)
GAPDH F	5'-gccaaggtgatccatgacaac-3'	498
GAPDH R	5'-gtccaccaccctgttgctgta-3'	
IL-8 F	5'-atgacttccaagctggccgtggct-3'	289
IL-8 R	5'-tctcagccctcttcaaaaacttctc-3'	
IL-6 F	5'-atgaactccttctccacaagcgc-3'	628
IL-6 R	5'-gaagagccctcaggctggactg-3'	
TNF-α F	5'-cgggacgtggagctggccgaggag-3'	355
TNF-α R	5'-caccagctggttatctctcagctc-3'	
IL-1β F	5'-ggcagactcaaattccagct-3'	249
IL-1β R	5'-ggacaggatatggagcaaca-3'	

The gel OD of the products were measured using image J software (http://imagej.nih.gov/ij/), OD values for cytokine genes were normalized with respect to the corresponding GAPDH OD values and fold change in gene expression for HCV NS3 treated cells were calculated with respect to the untreated control.

### ELISA

IL-8, IL-6, TNF-α, IL-1β and IL-10 ELISA kits were from eBiosciences (California, USA). After different treatment conditions, cell culture supernatants were collected and ELISA was performed as per the manufacturer's instruction.

### Flow cytometry

The cells were trypsinized after various treatment conditions and washed with DMEM and then with 1XPBS. The cells were fixed with 4% paraformaldehyde followed by incubation in FACS (flouorescent activated cell sorting) staining buffer (1XPBS with 3% FBS and 0.09% sodium azide) with 0.01% Triton X-100 15 minutes. The cells were stained with primary antibodies for MyD88, pNF-kB, TLR2, TLR6 and appropriate isotype control antibodies diluted in FACS staining buffer for 45 minutes on ice. Following 3 washes with FACS staining buffer, the cells were stained with FITC-conjugated secondary antibodies for 30 minutes on ice. The cells were washed 3 times with 1XPBS and analyzed in FACS Calibur. For all the conditions 10000 events were acquired and gated with respect to the unstained controls, results were expressed as % Gated cells.

### TLR2 real time PCR

Total cellular RNA was extracted from the cells using TRIzol (Ambion, Carlsbad, USA). First strand complementary DNA (cDNA) was synthesized by using oligo dT primers (Fermentas, USA) with 1 μg RNA and sensiscript RT (Qiagen, Hilden, Germany) as per the manufacture's protocol. Real time PCR was performed for 18S, and TLR2 genes. Primers are listed in table 2. PCR was amplified by quantiTect SYBR green master mix (Qiagen, Hilden, Germany) on rotor gene 3000 (Germany) machines. Relative gene expression levels were calculated by normalizing with corresponding 18S transcript levels and expressed as relative fold change compared with control cells.

**Table 2:** Real time PCR primer list.

Genes	Primers
18S F	5'-catggtgaccacgggtgac-3'
18S R	5'-ttccttggatgtggtagccg-3'
TLR2 F	5'-ggccagcaaattacctgtgtg-3'
TLR2 R	5'-aggcggacatcctgaacct-3'

### TLR2 blocking

Microglial cells were seeded onto 12 well plates ar a seeding density of ~8 X 10^4^ cells per well. Twenty four hrs post seeding, the cells were incubated with anti-TLR2 /Isotype control antibody at a concentration of 10 μg/ml for 2 hrs at 37°C followed by NS3 exposure. Anti-TLR2 was from Santacruz biotechnology (TLR2 (A-9): sc-166900).

### TLR6 silencing

Around 1.2 X 10^6^ cells were seeded on to 6 well plates. After 24 hrs post seeding, growth medium was changed. siRNAs were transfected (final concentration 100nM/ well) using Hiperfect lipofectamine reagent (Qiagen, Hilden, Germany) as per the manufacturer's instructions. Growth medium was replaced by fresh medium after 8 hrs and the cells were collected forFACS at 24 and 48 hr post transfection.

### Statistics

Student's t-test or One way analysis of variance with Bonferoni *post hoc* test was performed and p < 0.05 was considered significant.

## Results

### HCV NS3 induced pro inflammatory cytokine production in microglia

**Fig 1:**
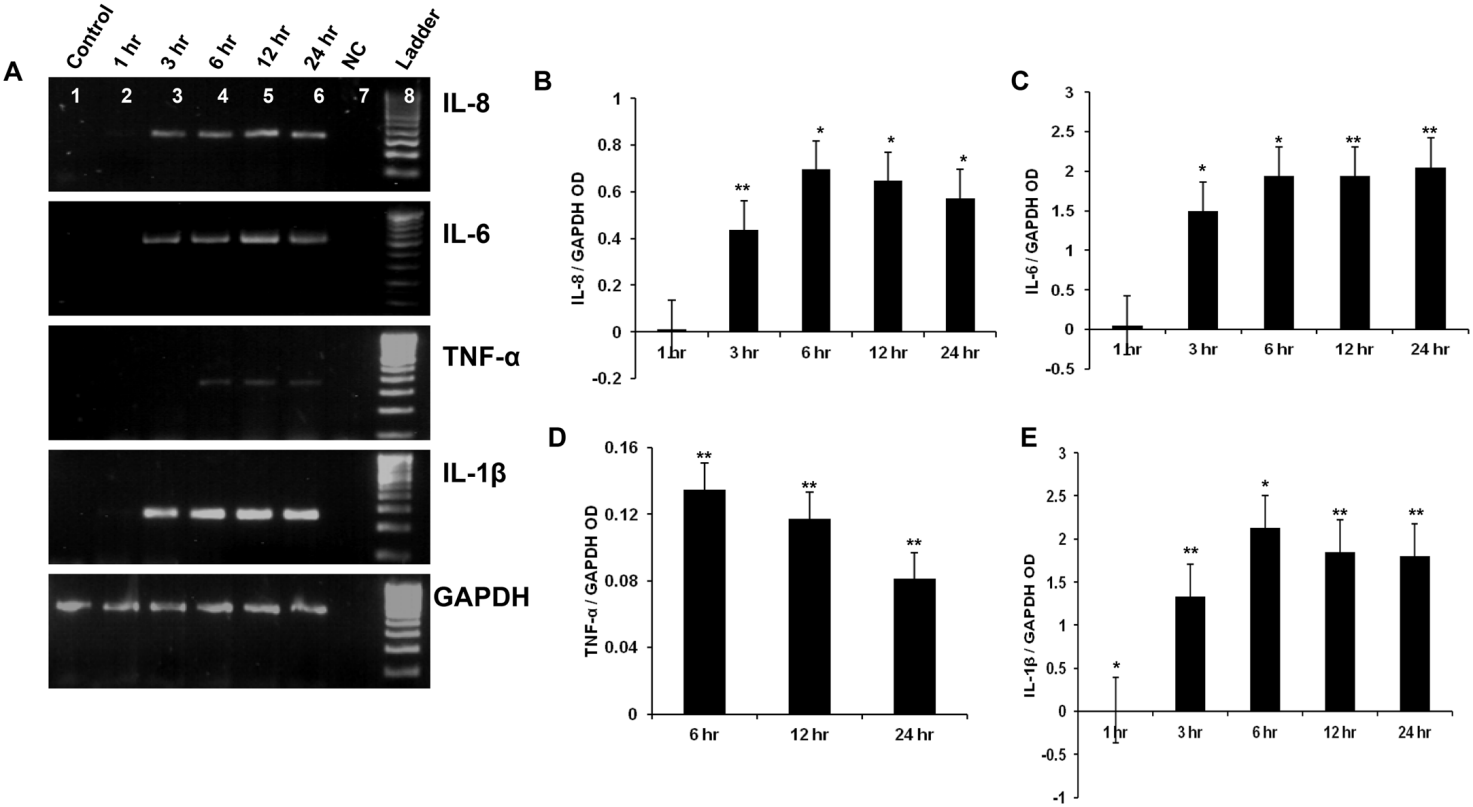
HCV NS3 induced pro inflammatory cytokine gene expression in microglia. CHME3 cells were exposed to 20 ng/ml of HCV NS3 for different time points and RT-PCR was performed to detect the cytokine gene expression at various time points. **(A)** IL-8, IL-6, TNF-α, IL-1β mRNA was expressed in the microglial cells exposed to NS3. The gel OD values were measured by image J and were expressed as cytokine OD values normalized to that of the corresponding GAPDH. **(B-D)** Microglia expressed IL-8 mRNA from 3 hr to 24 hr, **(C)** IL-6 mRNA was expressed from 3 hr to 24 hr, **(D)** TNF-α was expressed from 6 hr to 24 hr and **(D)** IL-1β was expressed from 3 hr to 24 hr. Lanes 1–8 represents control, NS3 treated cells at 1 hr, 3 hr, 6 hr, 12 hr, 24 hr, negative control (NC), 100 bp ladder in that order. The data is expressed as mean (n = 3) ± SE, * p < 0.05, ** p < 0.01.

**Fig 2:**
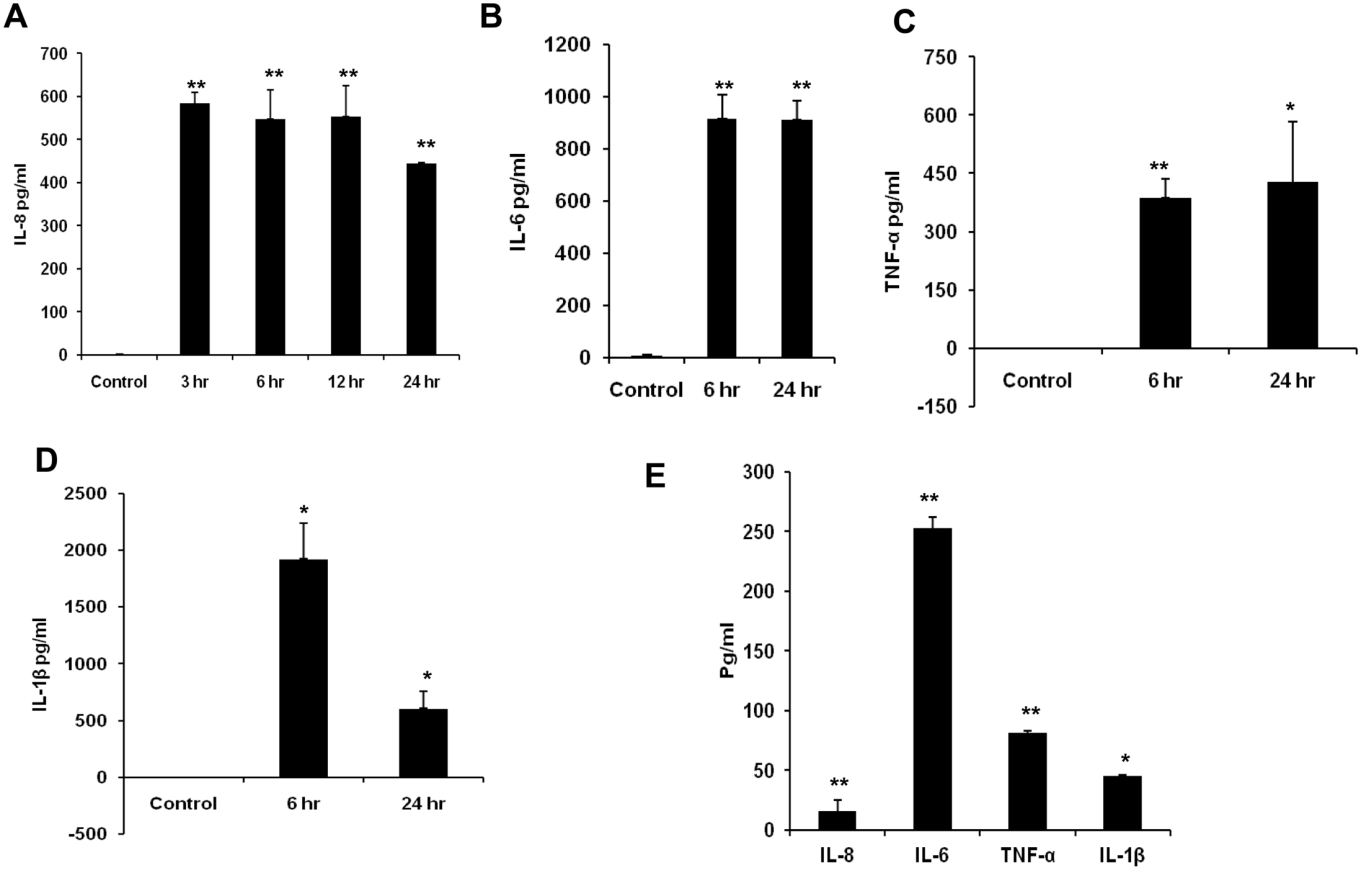
HCV NS3 induced pro inflammatory cytokine secretion by microglia. CHME3 cells were exposed to 20 ng/ml of NS3 for different time points and cell culture supernatant was collected for pro inflammatory cytokine ELISA. **(A)** IL-8 secretion was detected from 3 hr to 24 hr, **(B, C, D)** IL-6, TNF-α and IL-1βwas detected at 6 and 24 hr time points. The cells were exposed to 2 ng/ml of NS3 for 6 hrs and ELISA was performed for cytokines. **(E)** The cells secreted significant amount of IL-8, IL-6, TNF-α and IL-1β. The data is expressed as mean (n = 3) ± SE, * p < 0.05, ** p < 0.01.

Microglial cells were exposed to 20 ng/ml of HCV NS3 for 1, 3, 6, 12, and 24 hr time points. Within 3 hrs of exposure to NS3, microglia expressed IL-8 and IL-6 genes, while TNF-α expression was detected at only 6 hrtime point. IL-1β gene was expressed at 3 hr time point (Fig 1A). OD values of the PCR products were measured by image J and plotted as normalized OD values with that of the corresponding GAPDH OD. Semi quantitative expression of IL-8 gene indicated that the OD values reached maximum at 6 hr time point and this was almost sustained up to 24 hrs (Fig 1B), similar expression pattern was also observed for IL-6 gene (Fig 1C). The OD value for TNF-α gene was maximum at 6 hr time point followed by a time dependent decrease (Fig 1D). IL-1β gene expression increased in a time dependent manner from 3 hr to 6 hr followed by a sustained response for up to 24 hrs (Fig 1E). The cytokine protein expression was analysed by ELISA. Up on exposure to NS3, microglial cells secreted IL-8 chemokine at a concentration of ~ 500 pg/ml at 3, 6, 12 and 24 hr time points (Fig 2A). Similarly IL-6 was secreted at a concentration of ~ 900 pg/ml at 6 and 24 hr time points. TNF-α was detected at a concentration of ~300 pg/ml at 6 hr and ~400 pg/ml at 24 hr (Fig 2C). Compared to other pro inflammatory cytokines, IL-1β was secreted at a higher concentration. At 6 and 24 hr time points ~ 1200 pg/ml, ~ 900 pg/ml of IL-1β was secreted into the culture medium (Fig 2D). Our semi-quantitative RT-PCR data for cytokines (Fig 1B, 1C, 1D, and 1E) correlated with ELISA results. As we observed higher OD values for IL-1β and IL-6 compared to IL-8 and which was followed by TNF-α. Further we looked for the presence of cytokines in the cell culture medium exposed to 2ng/ml of NS3. There was a significant secretion of all the four cytokines at 6 hr exposure which indicates that NS3 mediates dose dependent cytokine secretion in microglia (Fig 2E). These results proved that NS3 was capable of inducing many pro inflammatory cytokine secretion in microglia which can in turn contribute towards neuroinflammation.

### Microglia signals via TLR2/TLR6 but not TLR1 during HCV NS3 mediated inflammation

**Fig 3:**
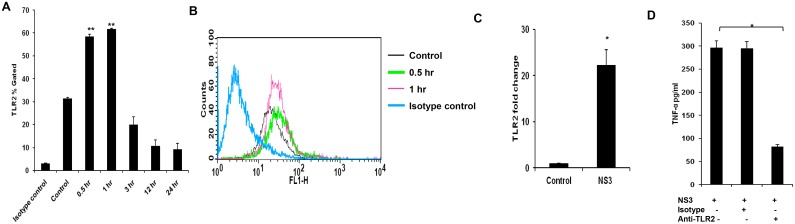
HCV NS3 mediated immune response via TLR2 CHME3 cells were exposed to 20 ng/ml of NS3 for different time points, cells were stained with TLR2 specific antibodies . Flow cytometry and immunofluorescence staining was performed to detect the cellular expression of the protein. Real time PCR was performed to detect TLR2 gene exxpression. **(A-B)** TLR2 expression was up regulated from 0.5 hr to 3 hr. **(C)** TLR2 transcripts were up regulated at 0.5 hr. **((D)** anti-TLR2 treatment down regulated the TNF-α secretion and the isotype control had no negative effect. Data is expressed as mean (n = 3) ± SE. * p < 0.05.

**Fig 4:**
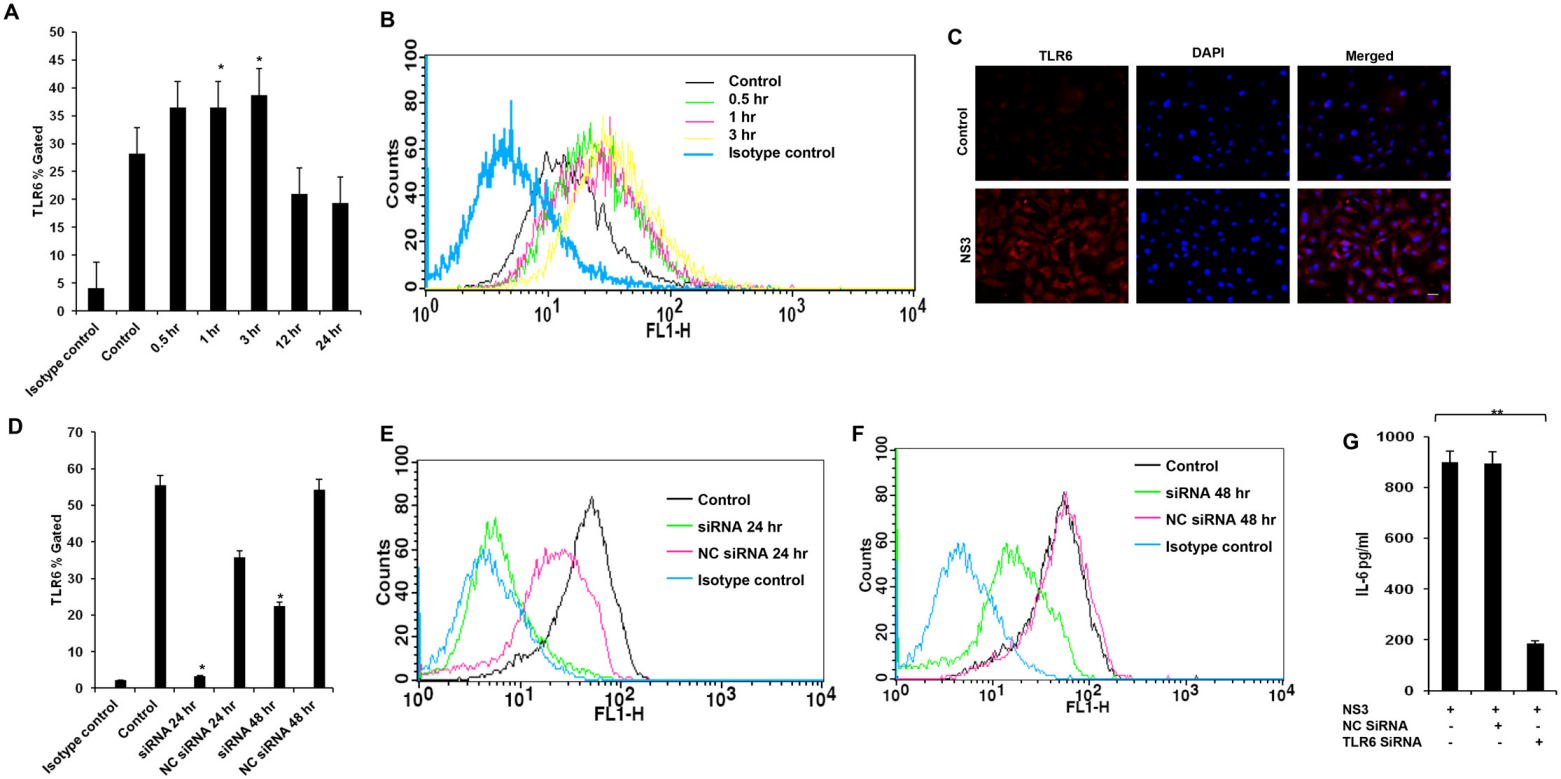
TLR6 was involved in NS3 mediated microglial activation. Microglia was exposed to 20 ng/ml of NS3 for different time points and cells were stained for TLR6 protein expression. **(A-B)** Flow cytometry results indicates that TLR6 expression was significantly high from 1 hr to 3 hr time point. **(C)** Also NS3 exposed microglia stained brighter for TLR6 at 3 hr time point compared to the control cells. **(D-F)** TLR6 expression was silenced by TLR6 specific siRNA and the **(G)** TLR6 silenced cells secreted significantly less IL-6. Data is expressed as mean (n = 3) ± SE. * p < 0.05. Scale bar corresponds to 50 micron.

**Fig 5:**
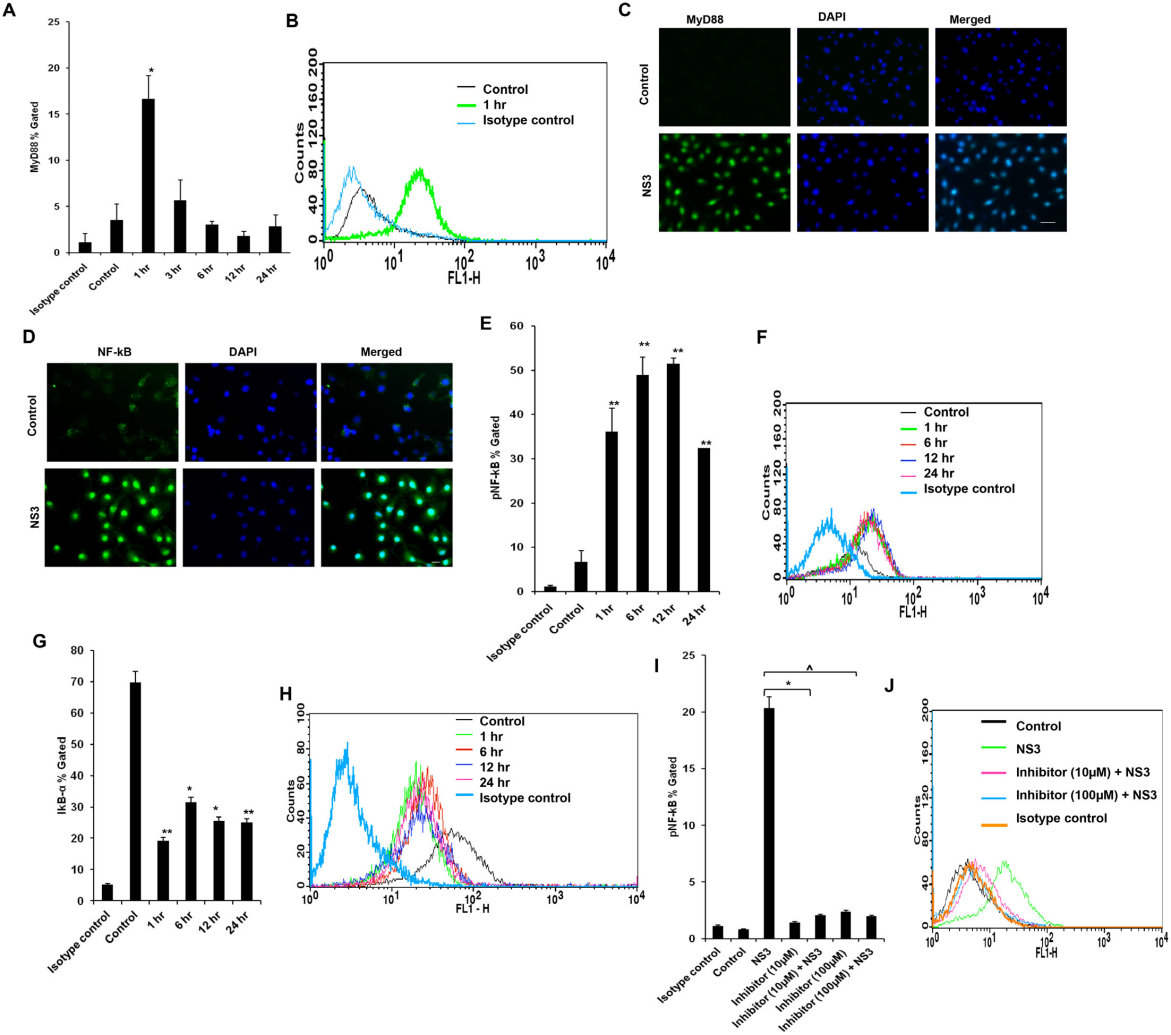
TLRs signalled via MyD88/NF-kB pathway. **(A-B)** MyD88 was up regulated only during 1 hr time point and there was no significant difference observed at later time points. **(C)** Fluorescent signal for MyD88 was visually high in NS3 exposed microglia at 1 hr time point. **(D)** Nuclear translocation of NF-kB was observed at 1 hr time point for NS3 exposed cells. Flow cytometry data shows that **(E-F)** pNF-kB protein expression was significantly upregulated and **(G-H)** IkB-α protein expression was significantly downregulated from 1 hr to 24 hr exposure to NS3. **(I-J)** CHME3 cells were pre-treated with NF-kB inhibitor, Ro 106–9920 for 16 hrs before being exposed to 20 ng/ml of NS3. Flow cytometry data shows that pNF-kB expression in 10 μM and 100 μM NF-kB inhibitor treated microglia were significantly less during NS3 exposure at 6 hr time point compared to NS3 alone exposed cells. Immunofluorescence data is representative of 3 independent experiments, scale bar represents 50 micron. The data is expressed as mean (n = 3) ± SE, * p < 0.05, ^ p < 0.05.

We studied the signalling mechanisms leading to the pro inflammatory cytokine secretion in microglial cells. The microglial cells were exposed to 20 ng/ml of NS3 at various time points and TLR1, TLR2 and TLR6 responses were measured. On 0.5 hr exposure to NS3, TLR2 protein expression was significantly up regulated (P < 0.01) in the microglial cells and the same trend was observed up to 3 hr (Fig 3A and 3B). This was revalidated by measuring TLR2 transcripts during NS3 treatment. TLR2 mRNA expression increased ~ 22 fold during 0.5 hr exposure to NS3 (Fig 3C). TLR6 protein expression was significantly high (p<0.05) at 1 hr and 3 hr time points. There was a increase in fluorescent intensity for 0.5 hr time point sample compared to the control however the p value for the same was 0.052(Fig 4A and 4B). TLR6 involvement was again confirmed by immunoflouorescence staining at 3 hr time point (Fig 4C). There was no significant difference in TLR1 expression (S2 Fig). To re-confirm the involvement of TLRs, blocking/gene silencing experiments were performed. Microglial cells blocked with anti-TLR antibody secreted significantly less TNF-α compared to NS3 exposed cell control (p<0.05) (Fig 3D). We silenced the TLR6 protein expression by SiRNA technology (Fig 4D, 4E and 4F). TLR6 silencing had a significantly negative impact (p<0.01) on IL-6 secretion during NS3 exposure compared to NS3 control (Fig 4G). These results indicates that both TLR2 and TLR6 acted as NS3 recognition receptors in microglia. Further TLRs signalling via MyD88/NF-kB pathway was studied. MyD88 protein expression was significantly up regulated at 1 hr time point (p < 0.05), from 3 hr to 24hrs there was no significant difference noted with respect to control (Fig 5A and 5B). NS3 treated cells stained fluuorescently bright compared to unteated control at 1 hr time point (Fig 5C). We captured the nuclear translocation of NF-kB at 1 hr time point (Fig 5D) followed by pNF-KB up regulation at 1, 3, 6, 12 and 24 hr time points (P < 0.01) (Fig 5E and 5F). IkB-α degradation was also observed at 1, 6, 12 and 24 hr time points (p<0.05) (Fig 5G and 5H). To re-confirm the NF-kB involvement, microglial cells were incubated with 100 μM and 10 μM concentrations of NF-kB inhibitor, Ro 106–9920 for overnight followed by 6 hr exposure to fresh growth medium containing NF-kB inhibitor at the same concentrations along with NS3 protein. pNF-kB expression was measured by flow cytometry and the results indicate that Ro 106–9920 at both the concentrations have effectively downregulated the protein expression compared to NS3 alone exposed cells (p<0.01) (Fig 5I). These data demonstrated the participation of TLR2 and TLR6 in recognizing NS3 and consecutive signalling via MyD88 adaptor protein and activation of NF-kB.

### NF-kB inhibitor down regulates NS3 mediated inflammation in microglia

**Fig 6:**
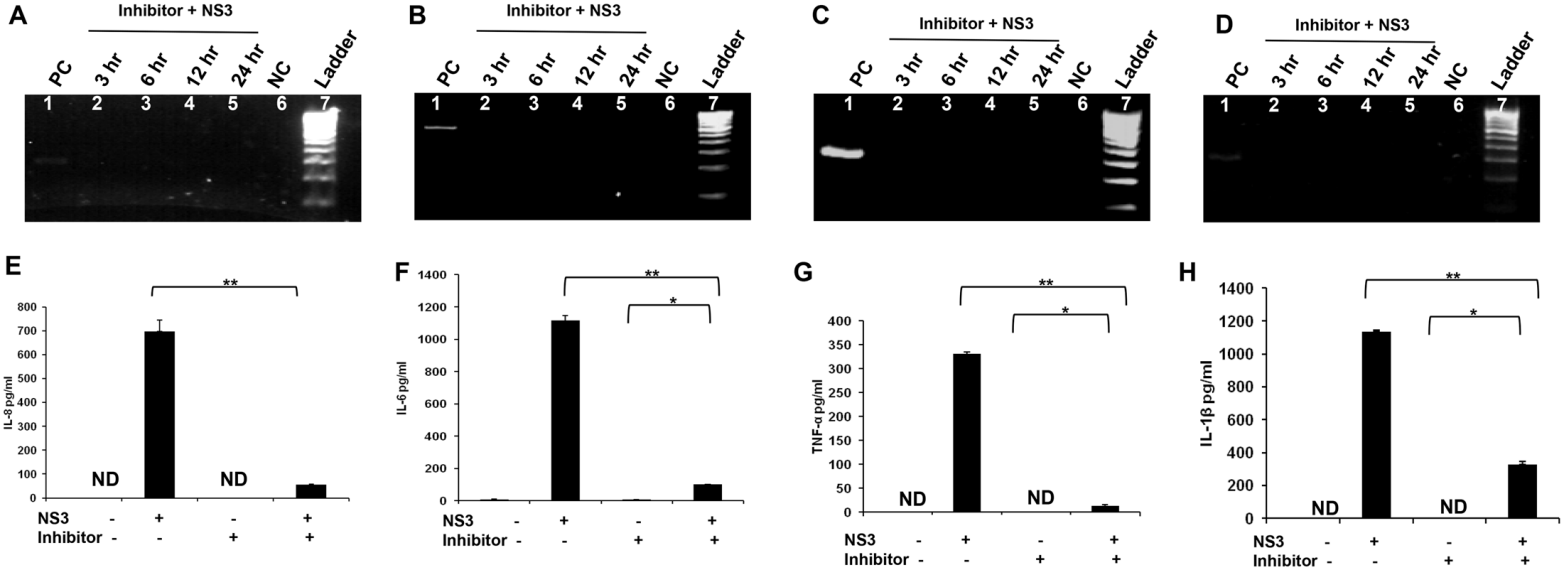
NF-KB inhibitor down regulates NS3 mediated inflammation in microglia. CHME3 cells were pre treated with NF-kB inhibitor, 10 μM Ro 106–9920 for 16 hrs before being exposed to 20 ng/ml of NS3. RT-PCR was performed for different time points and ELISA was performed for 6 hr time point to detect the cytokines. CHME3 exposed to 20 ng/ml of NS3 for 6 hrs were used as positive control. **(A, B, C and D)** IL-8, IL-6 and TNF-α expression was not detected and IL-1β gene band intensities were less in 3 hr to 24 hrs in the inhibitor treated cells when being exposed to NS3. NC represents the PCR reagent negative control. **(E, F, G and H)** IL-8, IL-6, TNF-α and IL-1β proteins were detected in the NS3 and inhibitor + NS3 treated cells and were absent in the control as well as inhibitor treated cells. There was a significant down regulation for IL-8 and other 3 cytokines in the inhibitor + NS3 treated cells compared to NS3 alone treated cells. PCR data is representative of 3 independent experiments. The data is expressed as mean (n = 3) ± SE, * p < 0.05, ** p < 0.01.

Since NF-kB was in an active state from 1 hr to 24 hr, and this transcription factor was involved in the induction of pro inflammatory cytokine gene expression, we hypothesised that inhibition of NF-kB might contribute towards controlling the pro inflammatory cytokine gene expression. Microglial cells were treated with NF-kB inhibitor Ro 106–9920 at a concentration of 10 μM (The lowest concentration which induced down regulation of pNF-kB in the previous experiment) overnight followed by incubation with fresh medium containing 10 μM Ro 106–9920 and 20 ng/ml of HCV NS3 for different time points. RT-PCR was performed to detect IL-8, IL-6, TNF-α and IL-1β gene expression. Microglia exposed to NS3 for 6 hr served as a positive control for the PCR reaction. When same amounts of the PCR products from positive control and NF-kB inhibitor treated cells were loaded on to the acrylamide gels, we could not detect IL-8, IL6 and TNF-α gene expression, IL-1β genes were expressed though the band intensities of the Ro 106–9920 treated cells were less compared to that of the positive control (Fig 6A, 6B, 6C and 6D) while the GAPDH was expressed normally in all the samples (S3 Fig). The cytokine secretion at 6 hr time point was measured by ELISA. The NF-kB inhibitor as such was not capable of eliciting IL-8 or other cytokine response. As explained earlier, microglia responded by secreting all the 4 cytokines at 6 hr exposure to NS3. IL-8 and other 3 cytokine secretions decreased when microglial cells were pretreated with NF-kB inhibitor before being exposed to NS3 along with pNF-kB inhibitor (Fig 6E, 6F, 6G and 6H). These results point out the role of NF-kB in mediating microglial inflammation associated with HCV NS3.

### TLR agonist induced immune protection against NS3 mediated inflammation

**Fig 7:**
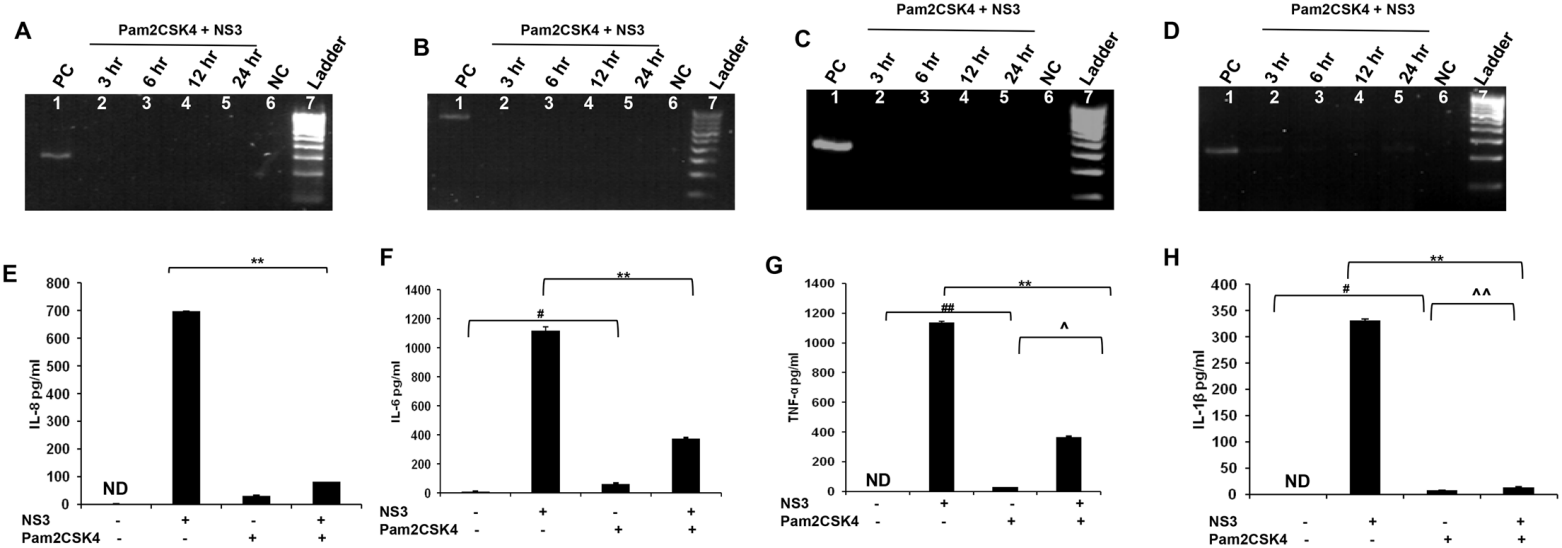
Pam2CSK4 induced immune tolerance against NS3 mediated inflammation. The microglial cells were treated with 50 ng/ml of Pam2CSK4 for 16 hrs. The medium was replaced with fresh growth medium with or without NS3 for another 6 hours. RT-PCR and ELISA was performed to detect cytokine gene and protein expression respectively. 6 hr NS3 exposed microglia served as positive control. **(A, B, C and D)** Pam2Csk3 treatment completely blocked IL-8, IL-6 and TNF-α expression. IL-1β expression was detected however the band intensities were less compared to that of the positive control. NC represents the PCR reagent negative control. **(E, F, G and H)** ELISA results shows that IL-6, TNF-α and IL-1β were secreted at a significant level in the Pam2CSK4 treated cells even after replacing the cells with fresh growth medium without agonist (# p < 0.05, ## p< 0.01). TNF-α and IL-1β were significantly up regulated in Pam2CSK4 + NS3 treated cells compared to Pam2CSK4 exposed cells (^ p < 0.05, ^^ p < 0.01). There was a significant down regulation for IL-8 and other 3 cytokines in the Pam2CSK4 + NS3 treated cells compared to NS3 alone treated cells (* p < 0.05, ** p < 0.01). PCR data is representative of 3 independent experiments. The data is expressed as mean (n = 3) ± SE.

**Fig 8:**
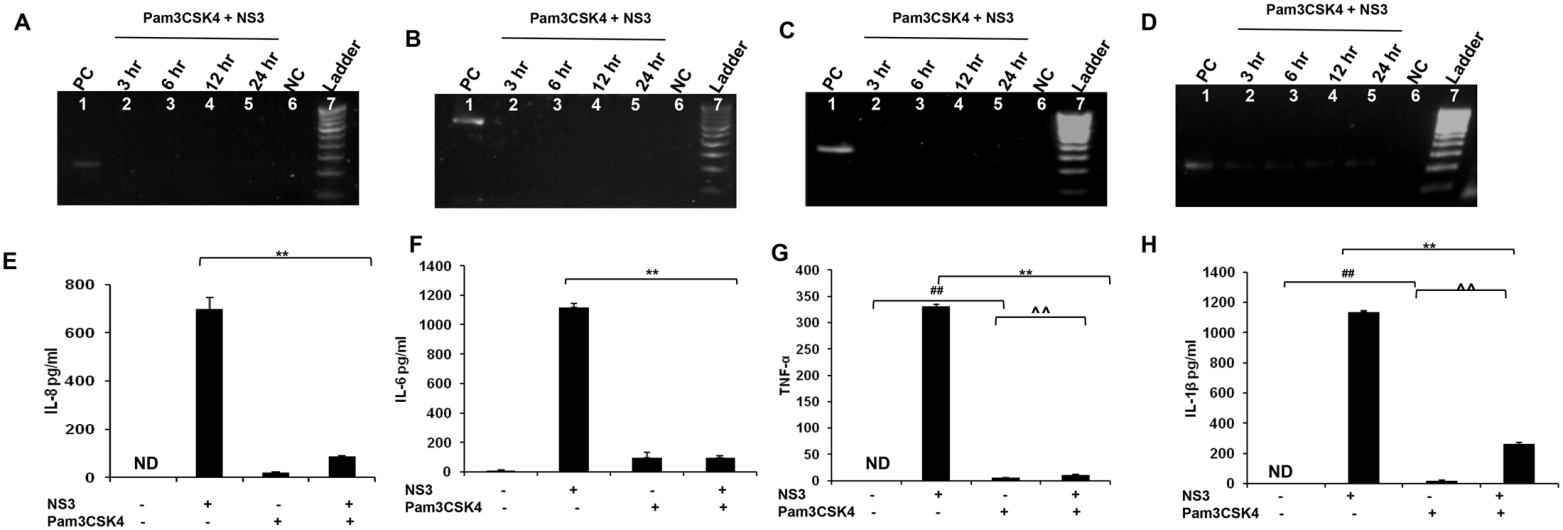
Pam3CSK4 induced immune tolerance against HCV NS3 mediated inflammation. Microglia was pre treated with 50 ng/ml of Pam3CSK4 for 16 hrs. RT-PCR and ELISA was performed to study the immune tolerance mediated by Pam3CSK4. **(A, B, C and D)** Pam3CSK4 treatment completely blocked IL-8, IL-6 and TNF-α gene expression and IL-1β gene expression was down regulated compared to positive control (PC NC represents the PCR reagent negative control. **(E, F, G and H)** TNF-α and IL-1β were secreted at a significant level in the Pam3CSK4 treated cells even after replacing the cells with fresh growth medium without agonist (## p< 0.01). TNF-α and IL-1β were significantly upregulated in Pam3CSK4 + NS3 treated cells compared to Pam3CSK4 exposed cells (^ p < 0.05, ^^ p < 0.01). There was a significant down regulation for IL-8 and other 3 cytokines in the Pam3CSK4 + NS3 treated cells compared to NS3 alone treated cells (** p < 0.01). The data is expressed as mean (n = 3) ± SE.

**Fig 9:**
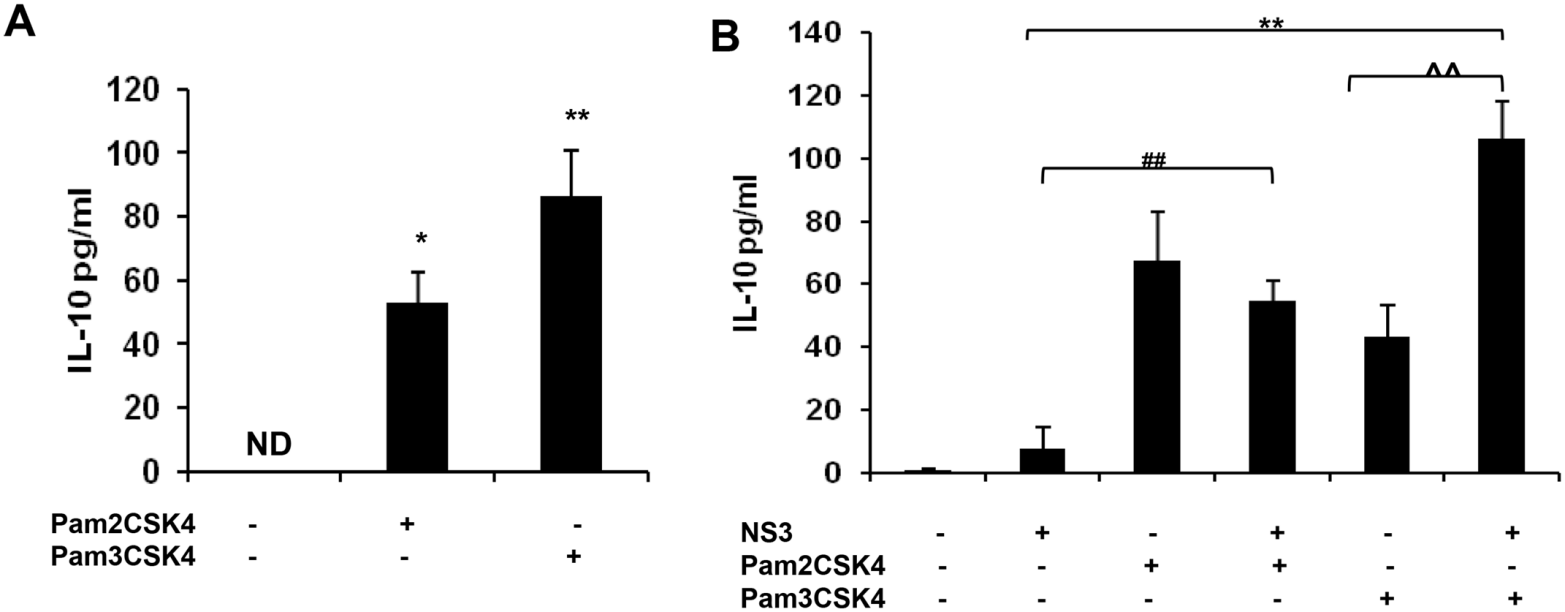
TLR agonist treatment induced IL-10 production in microglia. The microglial cells were treated with 50 ng/ml of Pam2CSK4 or Pam3CSK4 for 16 hrs. The medium was replaced with fresh growth medium with or without NS3 for another 6 hours. ELISA was performed to detect IL-10 secretion. CHME3 exposed to 20 ng/ml of NS3 for 6 hrs were used as positive control. **(A)** Microglial cells exposed to Pam2CSK4 or Pam3CSK4 for 16 hrs secreted IL-10 at a significant level (* p < 0.05). **(B)** IL-10 production was significantly up regulated in Pam2CSK+NS3 (** p < 0.01) and Pam3CSK4+NS3 (## p < 0.01) treated cells compared to NS3 alone treated cells, also IL-10 significantly up regulated in Pam3CSK4+NS3 exposed cells compared to Pam3CSK4 exposed cells (^^ p < 0.01). The data is expressed as mean (n = 3) ± SE.

**Fig 10:**
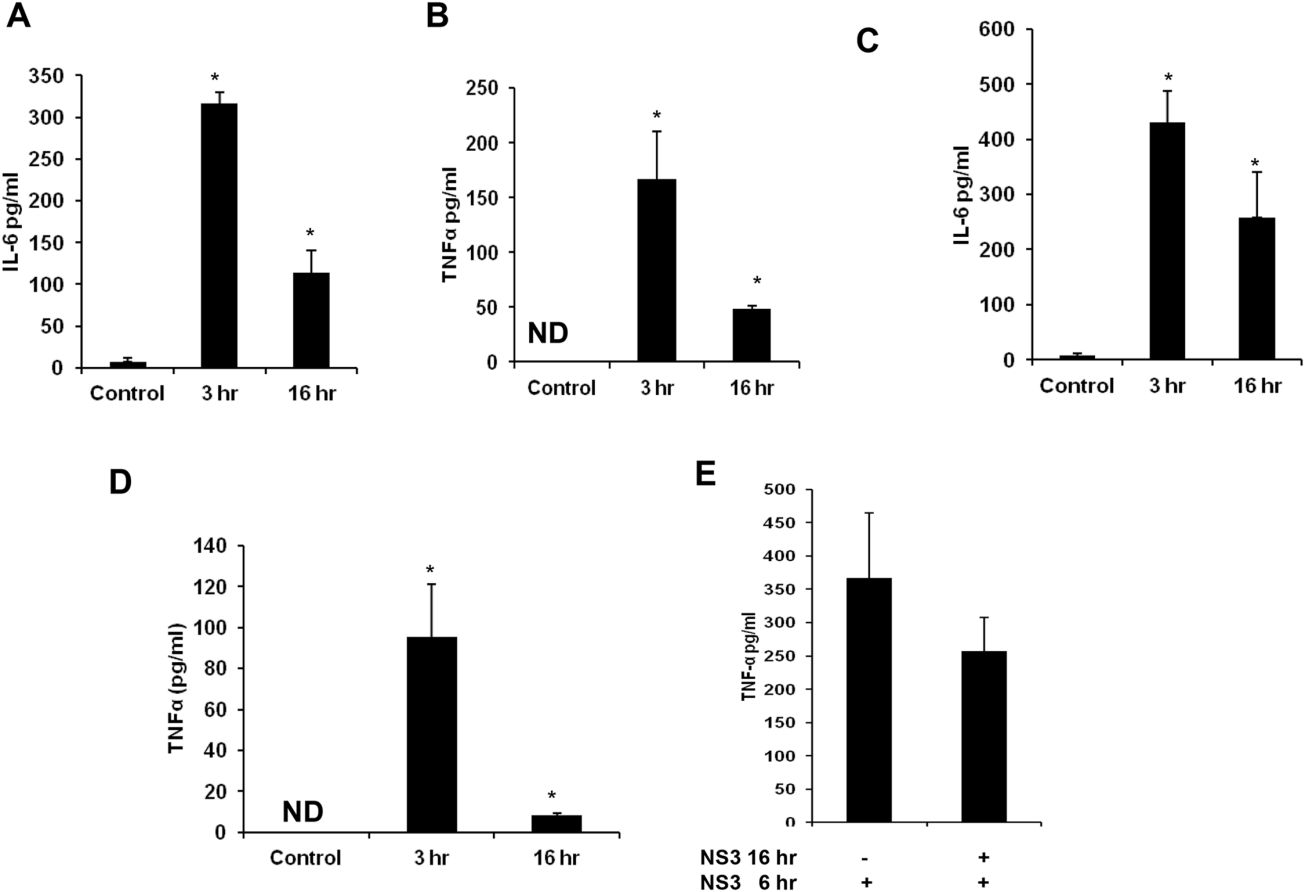
TLR agonist treatment induced pro-inflammatory cytokines and NS3 pre-treatment does not induce immune tolerance. Microglia was exposed to 50 ng/ml of Pam2CSK4 and Pam3CSK4 and pro-inflammatory cytokine expressions were measured by ELISA. **(A and B)** Pam2CSK4 treatment induced IL-6 and TNF-α production at 3 and 16 hr time point. **(C and D)** Similarly Pam3CSK4 treatment induced IL-6 and TNF-α production. Microglial cells were pre-treated with 20 ng/ml of NS3 protein for 16 hrs before 6 hr NS3 treatment to study the immune tolerance induction by NS3. **(E)** There was no significant difference observed for TNF-α production inNS3 exposed and NS3 pre-treated cells. The data is expressed as mean (n = 3) ± SE, *p< 0.05.

Earlier reports indicates the role of TLR agonist in mediating immune protection in many inflammatory conditions [16]. Since mciroglia responded to NS3 via TLR2 and TLR2 may mediate the signalling along with TLR1 or TLR6 which can act as co-receptors, the microglial cells were pre treated with Pam2CSK4 which is a TLR2/6 agonist or with Pam3CSK4 which is a TLR2/1 agonist. Both the TLR agonists were dissolved in endotoxin free water and hence there was no vehicle controls were included in the experiments. RT-PCR and ELISA was performed for IL-8, IL-6, TNF-α and IL-1β. CHME3 exposed to NS3 for 6 hrs was used as positive control in the RT-PCR experiment. For IL-8, IL-6 and TNF-α, we could not capture the gene expression at all the time points in the Pam2CSK4 and Pam3CSK4 treated microglia (Figs 7A, 7B, 7C, 8A, 8B and 8C). Both the agonist treated microglia expressed IL-1β gene during NS3 exposure at different time points, however the band intensities were less compared to that of the positive control (Figs 7D and 8D) while the GAPDH was expressed normally in all the samples (S4 Fig and S5 Fig). ELISA results indicates that both the agonist treatments had a negative effect upon all the cytokine secretion during NS3 exposure. All the four cytokine concentration significantly down regulated (p < 0.05) in the Pam2CSK4 and Pam3CSK4 conditioned microglia compared to the untreated microglia at 6 hr exposure to NS3 (Figs 7E, 7F, 7G, 7H, 8E, 8F, 8G and 8H). However Pam2CSK4 and Pam3CSK4 conditioned microglia secreted ~ 30 pg/ml and ~ 20 pg/ml of IL-8; ~ 60 pg/ml and 90 pg/ml IL-6; ~ 20 pg/ml and 10 pg/ml TNF-α; ~ 20pg/ml and ~ 30 pg/ml IL-1β respectively (P < 0.05) (Figs 7E, 7F, 7G, 7H, 8E, 8F, 8G and 8H). We hypothesised the role of anti inflammatory cytokines behind microglial negative response against NS3 during TLR agonist treatment. To prove this we measured IL-10 secretion by ELISA. In the control samples we could not detect IL-10, while microglia exposed to NS3 for 6 hr secreted very low amount of IL-10 (~ 7 pg/ml). After 16 hrs of treatment with Pam2CSK4 and Pam3CSK4, the microglial culture medium was analysed for IL-10 secretion. Both Pam2CSK4 and Pam3CSK4 treatments induced ~ 52 pg/ml and ~ 86 pg/ml of IL-10 respectively (Fig 9A). Further the conditioned mediums were removed and replaced with fresh growth medium or growth medium with 20 ng/ml NS3. After 6 hrs of treatment, the culture mediums were collected and ELISA was performed for IL-10. Both Pam2CSK4 and Pam3CSK4 conditioned microglia secreted ~ 60 pg/ml and ~ 40 pg/ml of IL-10 even after being replaced with fresh mediums without TLR agonists. At the same time NS3 exposed microglia which were pre treated with Pam2CSK4 or Pam3CSK4 were also expressing ~ 50 pg/ml and ~ 100 pg/ml respectively which were significantly higher (p < 0.05) than that of the TLR agonist untreated microglia exposed to NS3 (Fig 9B). These results hinds that TLR agonists Pam2CSK4 and Pam3CSK4 conditioning induces IL-10 secretion which could be the possible mechanism involved in down regulating cytokine secretion during NS3 exposure. Other than IL-10 secretion, both Pam2CSK4 and Pam3CSK4 treatments induced IL-6 and TNF-α production at 3 and 16 hr time points (p<0.05) (Fig 10A, 10B, 10C and 10D). We further looked for the possible immune tolerance induced by NS3 protein itself. For this study, microglial cell were pre-exposed to 20 ng/ml of NS3 before being treated with NS3 for another 6 hr. We could not find any significant difference in the cytokine production among NS3 pre-treated as well as untreated microglia (Fig 10E).

## Discussion

Emerging evidences points the neuroinvasion of HCV [4]. *In vivo* studies have documented the presence of HCV NS3 protein in microglia [24]. In this context our study aims to find out the neuroinflammatory potential of NS3 in an *in vitro* condition. Cell culture derived HCV was capable of infecting human fetal microglia and astrocytes *in vitro* and recombinant HCV core was capable of inducing IL-1β and TNF-α secretion in microglia but not astrocytes where as IL-8 was secreted by both the cell types [25]. HCV core induced CXCL10 and CXCL8 mRNA expression was highest at 6 hr time point and quenched at 24 hr time point however CXCL8 protein was detected only after 12 hr exposure. In our study NS3 was capable of activating pro inflammatory cytokine gene expression within 3 hr time point. TNF-α was the last among the four cytokines expressed with respect to NS3 exposure, as the mRNA expression was detected only from 6 hr time point.

TLR2 and its cellular co receptors are involved in the cellular activation mediated by NS3 in human and mouse cells [26] also NS3 mediated IL-10 and TNF-α production by human monocyte derived macrophages. We could link the activation of a major transcription factor NF-kB within 1 hr time point via TLR2 or TLR6 as there was an increased expression of TLR2, TLR6, MyD88 and pNF-kB. Though we could detect the increased expression of TLR2, TLR6 and pNF-kB at later time points, MyD88 was up regulated only during the early phase of the immune response. This clearly shows that TLR2/MyD88/NF-kB pathway was involved in the innate immune response in microglia. Though TLR2, TLR6 and pNF-kB proteins were up regulated in the later time points, MyD88 was up regulated only during early phase of the immune response, we assume that the immune activation signals are passed via MyD88 at the early phase. In connection to this hypothesis, participation of TLR1 and TLR6 as co receptors in NS3 mediated macrophage activation and innate immunity has been observed in humans [26]. Cell surface expressed TLR6 along with TLR2 was required for the activation of *Mycoplasma* derived molecular patterns [27]. There might be possible independent signalling of TLR2 and TLR6 in NS3 mediated microglial inflammation as at 30 minute time point we could detect only the significant up regulation of TLR2 and TLR6 was up regulated only at 1 hr time point.

In our study we find out that NF-kB is the major transcription factor involved in NS3 mediated inflammation in microglia as NF-kB inhibition had a dramatic effect upon the steady state levels of all the four pro inflammatory cytokine mRNA as well as proteins. NF-kB inhibitor Ro 106–9920 has been reported to block lipopolysaccharide induced TNF-α, IL-1β and IL-6 in peripheral blood monocytes via blocking IKappa B(alpha) degradation and NF-kB activation [28]. TLR ligand stimulated human macrophages and dendritic cells exhibited cytokine expression via TLR mediated NF-kB, MAPKs and PI-3K pathways and pharmacological inhibitors of these pathways significantly inhibited cytokine mRNA and protein expression [29]. Similarly blockade of IL-15 via inhibiting NF-kB pathway has down regulated the cytokine and chemokine release by activated microglia [30]. Since the inhibitor was successful in partially inhibiting the cytokine production we assume that this could be explained by the involvement of multiple pathways regulating cytokine gene expression.

The role of TLR agonists as therapeutic agents against infectious diseases and associated innate immune regulation has been widely appreciated [31]. Pam2Cys induced innate immune response against influenza virus in mice characterised by increased expression of IL-10, IL-6, IFN-γ [32]. Since TLR1, TLR2 and TLR6 transcripts were expressed in CHME3 cells used in our study (Figs 3A, 4A and S2 Fig) we used Pam2CSK4 which mediated immune response via TLR2/1 and Pam3CSK4 which acts via TLR2/6 in our study. The TLR agonist concentration were chosen based on previous literature evidences [33]. Sixteen hour pre treatment with 50 ng/ml of Pam2CSK4 or Pam3CSK4 imparted immune tolerance in microglia against NS3. The cytokine responses were similar to that we had observed with respect to NF-kB inhibitor treatment. IL-8, IL-6, IL-1β and TNF-α down regulated to a significant level at the gene and protein level in Pam2CSK4 or Pam3CSK4 pre treated microglia during NS3 exposure. Partially this mechanism could be explained by the significant up regulation of an anti inflammatory cytokine IL-10 in TLR agonist treated cells. TLR agonist treated microglia secreted IL-10 at higher concentrations even 6 hrs after removing the TLR agonists. IL-10 secretion was sustained at the same concentration level by Pam2CSK4 and Pam3CSK4 exposed microglia treated with NS3 where as NS3 alone treated microglia secreted IL-10 at a concentration which is 10 fold lower. IL-10 is a potent inhibitor of cell-mediated inflammation and functions primarily via down regulating the expression of pro inflammatory cytokines mediating self tolerance [34]. Systemic injection of TLR2/6 agonist Pam2 lipopeptides has induced IL-10 production in mice models which prevented effective anti tumor immunity [35]. In our experimental condition the IL-10 production was found to be effective against down regulating the pro inflammatory immune response in microglia. Similarly human fetal microglial RANTES production was inhibited by IL-10 [36]. IL-10 knock down studies will further demonstrate the role of this cytokine in toll like receptor induced immune tolerance against NS3. However in our study both the TLR agonists induced IL-6 and TNF-α secretion at early and late time points (6 hr and 16 hr) which suggests the possible protective immune response mediated by TLR agonists via secretion of pro-inflammatory cytokines during active viral infection. Our current observation does not rule out the possible down regulation of TLR during TLR ligand induced immune protection and from the literature evidence we find that the molecular mechanisms contributing towards immune tolerance can be complex including the possible down regulation of the corresponding TLRs [37]. HCV core protein is known to mediate homotolerance [38]. However NS3 pre-treatment did not induce immune tolerance against NS3 in microglia since we could not detect any significant difference in TNF-α production by NS3 pre-treated microglia as well as naive microglia exposed to the viral protein. Also it is to be considered that our studies are limited by the use of cell line and hence it is very much possible the inflammatory response mediated by primary microglia may differ moderately. Previous studies have demonstrated the TLR2/6 or TLR1/6 co-receptor mediated cytokine expression in PBMCs [26]. However TLR1 was not found to be involved in the NS3 mediated inflammation in our study and this is the first study demonstrating TLR2 and TLR6 involvement in mediating immune response against NS3 in microglia which may be relevant in understanding the neuroinflammatory potential of this viral protein.

## Conclusion

In conclusion HCV NS3 protein was capable of inducing immune response in human microglia cell lines by inducing the secretion of pro inflammatory cytokines such as IL-8, IL-6, TNF-α and IL-1β. TLR2 and TLR6 acted as NS3 recognition receptors which activated MyD88/NF-kB pathway to mediate transcription of pro inflammatory cytokine genes. A synthetic NF-kB inhibitor Ro 106–9920 was found to be effective against down regulating NS3 mediated inflammation. Pre treatment of TLR agonists Pam2CSK4 and Pam3CSK4 mediated immune protection against NS3 mediated inflammation by secreting IL-10 in microglia. These immune modulation strategies could be implemented in controlling HCV mediated neuro inflammation. Further studies on other HCV antigens and the TLR responses will help us to understand the molecular mechanisms underlying HCV associated neuro inflammation.

## Supporting Information

S1 Fig Microglia marker studies. CD11b, a microglial marker was detected in the CHME3 cells used in our study. Fig A Real time amplification plot for CD11b and the primers used. Fig B The amplified product (Lane 1) was resolved in 3% agarose gel along with 100 bp DNA ladder (Lane 2).
(TIF)

S2 Fig NS3 does not alter TLR1 expression in microglia. CHME3 cells were exposed to 20 ng/ml of NS3 for different time points, cells were stained with TLR1 and flow cytometry was performed to detect the cellular expression of this protein. There was no significant difference in TLR1 for all the time points. The data is expressed as mean (n = 3) ± SE.
(TIF)

S3 Fig GAPDH PCR for NF-kB inhibitor treated cells. CHME3 cells were pre treated with NF-kB inhibitor, 10 μM Ro 106–9920 for 16 hrs before being exposed to 20 ng/ml of NS3. RT-PCR was performed for different time points. The GAPDH band intensities were visually same for all the samples. Lanes 1–7 represents positive control (PC), Inhibitor + NS3 treated cells at 3 hr, 6hr, 12 hr, 24 hr, negative control (NC), 100 bp ladder in that order. The data is representative of 3 independent experiments.
(TIF)

S4 Fig GAPDH PCR for Pam2CSK4 treated cells. The microglial cells were treated with 50 ng/ml of Pam2CSK4 for 16 hrs. The medium was replaced with fresh growth medium with NS3 for another 6 hours. RT-PCR was performed for different time points. The GAPDH band intensities were visually same for all the samples. positive control (PC), Pam2CSK4 + NS3 treated cells at 3 hr, 6hr, 12 hr, 24 hr, negative control (NC), 100 bp ladder in that order. The data is representative of 3 independent experiments.
(TIF)

S5 Fig GAPDH PCR for Pam3CSK4 treated cells. The microglial cells were treated with 50 ng/ml of Pam3CSK4 for 16 hrs. The medium was replaced with fresh growth medium with NS3 for another 6 hours. RT-PCR was performed for different time points. The GAPDH band intensities were visually same for all the samples. positive control (PC), Pam3CSK4 + NS3 treated cells at 3 hr, 6hr, 12 hr, 24 hr, negative control (NC), 100 bp ladder in that order. The data is representative of 3 independent experiments.
(TIF)
