# Supplementary figures and images for: Hepatitis C Virus NS3 Mediated Microglial Inflammation via TLR2/TLR6 MyD88/NF-κB Pathway and Toll Like Receptor Ligand Treatment Furnished Immune Tolerance
Source: PLoS One. 2015 May 12;10(5):e0125419. doi: 10.1371/journal.pone.0125419 (PMC4428696; doi:10.1371/journal.pone.0125419)

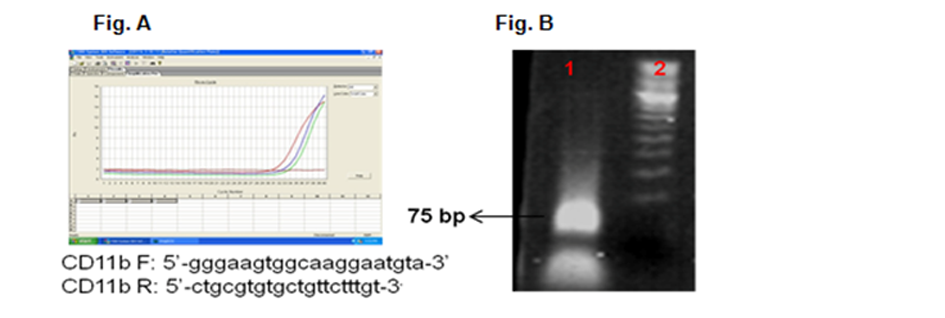

Supplement: S1 Fig — CD11b, a microglial marker was detected in the CHME3 cells used in our study. Fig A Real time amplification plot for CD11b and the primers used. Fig B The amplified product (Lane 1) was resolved in 3% agarose gel along with 100 bp DNA ladder (Lane 2). (TIF) [file pone.0125419.s001.tif]

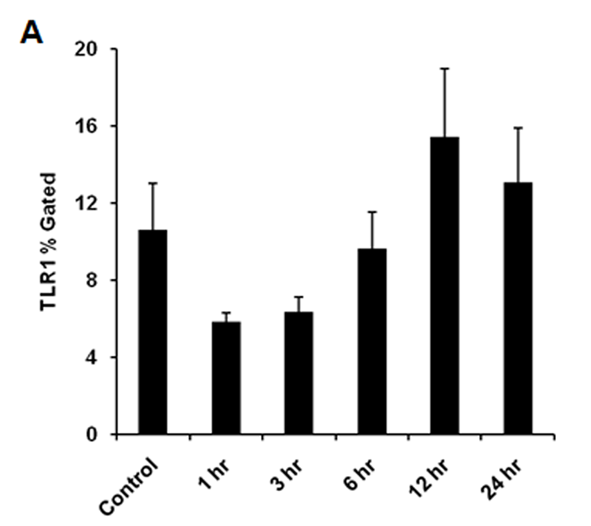

Supplement: S2 Fig — CHME3 cells were exposed to 20 ng/ml of NS3 for different time points, cells were stained with TLR1 and flow cytometry was performed to detect the cellular expression of this protein. There was no significant difference in TLR1 for all the time points. The data is expressed as mean (n = 3) ± SE. (TIF) [file pone.0125419.s002.tif]

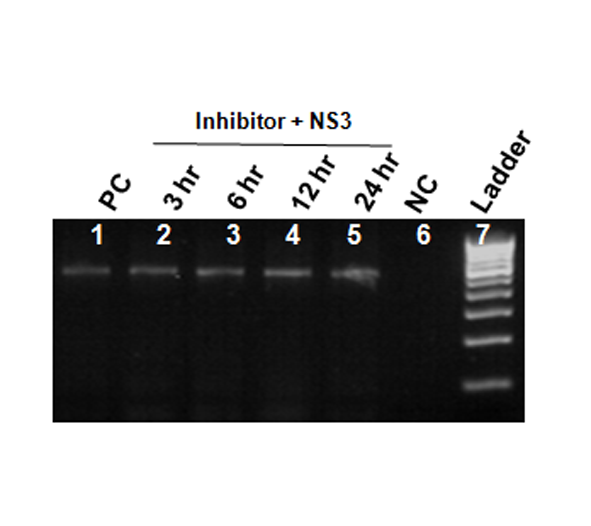

Supplement: S3 Fig — CHME3 cells were pre treated with NF-kB inhibitor, 10 μM Ro 106–9920 for 16 hrs before being exposed to 20 ng/ml of NS3. RT-PCR was performed for different time points. The GAPDH band intensities were visually same for all the samples. Lanes 1–7 represents positive control (PC), Inhibitor + NS3 treated cells at 3 hr, 6hr, 12 hr, 24 hr, negative control (NC), 100 bp ladder in that order. The data is representative of 3 independent experiments. (TIF) [file pone.0125419.s003.tif]

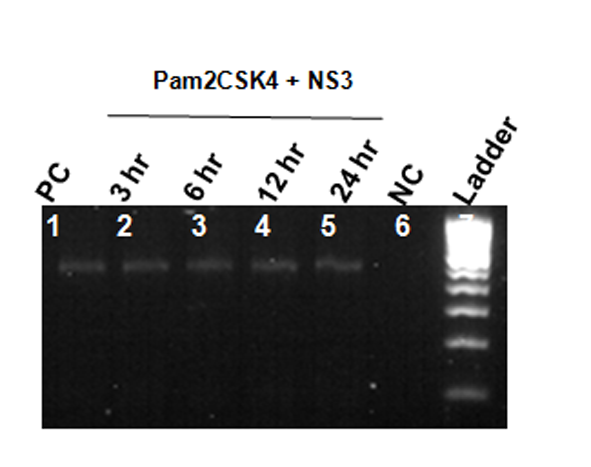

Supplement: S4 Fig — The microglial cells were treated with 50 ng/ml of Pam2CSK4 for 16 hrs. The medium was replaced with fresh growth medium with NS3 for another 6 hours. RT-PCR was performed for different time points. The GAPDH band intensities were visually same for all the samples. positive control (PC), Pam2CSK4 + NS3 treated cells at 3 hr, 6hr, 12 hr, 24 hr, negative control (NC), 100 bp ladder in that order. The data is representative of 3 independent experiments. (TIF) [file pone.0125419.s004.tif]

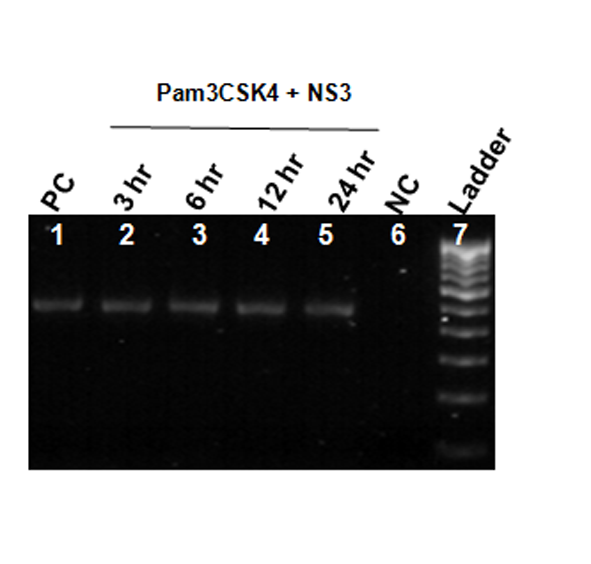

Supplement: S5 Fig — The microglial cells were treated with 50 ng/ml of Pam3CSK4 for 16 hrs. The medium was replaced with fresh growth medium with NS3 for another 6 hours. RT-PCR was performed for different time points. The GAPDH band intensities were visually same for all the samples. positive control (PC), Pam3CSK4 + NS3 treated cells at 3 hr, 6hr, 12 hr, 24 hr, negative control (NC), 100 bp ladder in that order. The data is representative of 3 independent experiments. (TIF) [file pone.0125419.s005.tif]
